# Disclosure of oral pre-exposure prophylaxis use for HIV prevention among women enrolled in a contraceptive study: qualitative findings from Durban, South Africa

**DOI:** 10.3389/fgwh.2024.1505643

**Published:** 2025-01-07

**Authors:** Ivana Beesham, Mags Beksinska, Cecilia Milford, Leila E. Mansoor

**Affiliations:** ^1^ Wits MatCH Research Unit (WMRU), Faculty of Health Sciences, University of the Witwatersrand, Durban, South Africa; ^2^ Centre for the AIDS Programme of Research in South Africa (CAPRISA), University of KwaZulu-Natal, Durban, South Africa

**Keywords:** oral pre-exposure prophylaxis, PrEP disclosure, young women, South Africa, reactions to PrEP disclosure

## Abstract

**Background:**

Disclosure of oral pre-exposure prophylaxis (PrEP) use for HIV prevention may improve adherence to PrEP; however, disclosure can be challenging and may result in stigma. Here, we describe disclosure of PrEP use among young women enrolled in a contraceptive study.

**Methods:**

In this qualitative study, we conducted semi-structured, in-depth, face-to-face interviews with 13 women aged 18–25 years who initiated oral PrEP for HIV prevention during the Evidence for Contraceptive Options and HIV Outcomes (ECHO) Trial. Interviews were conducted in 2021 with women from Durban, South Africa. In this analysis, we explore women's experiences of PrEP disclosure including whether they disclosed PrEP use, who they disclosed to and the reaction to disclosure, and the impact of disclosure on PrEP use. Interviews were conducted in English, audio-recorded and transcribed. Data were analysed thematically.

**Results:**

All women disclosed oral PrEP use to at least one individual, with some women disclosing to multiple individuals including family, friends, partners and community members. Few women did not disclose oral PrEP use to their partner due to anticipating a negative reaction from the partner, feeling that the partner would assume the woman has HIV and is taking antiretroviral therapy and fear that the partner would associate PrEP use with the woman having other partners. Reactions to oral PrEP disclosure were generally supportive or neutral, however, few women reported negative reactions that included distrust in the efficacy of PrEP to prevent HIV, discouraging the woman from using oral PrEP because of the assumption that PrEP is HIV treatment, and concern about the woman having oral PrEP side effects. Negative reactions to disclosure generally did not impact on oral PrEP use. Supportive disclosures sometimes resulted in reminders for oral PrEP dosing.

**Conclusion:**

Our findings indicate that women are willing to disclose their PrEP use to at least one other person when supported. These study findings may contribute to future PrEP counselling guidelines and strategies.

## Introduction

Oral tenofovir-based pre-exposure prophylaxis (PrEP) for HIV prevention was approved for use in South Africa in 2015 (1). Implementation began in 2016, in a staged manner starting with female sex workers (2) and then expanding to include additional populations such men who have sex with men, university students, adolescent girls and young women (AGYW) and the general population (3). HIV prevention options including daily oral PrEP are critical for AGYW as it is estimated that more than 90,000 women aged 15 years and over will acquire HIV in South Africa in 2024 (4). Adherence to daily oral PrEP is an important component of PrEP efficacy but has been challenging in many settings (5).

Disclosure of oral PrEP use may improve adherence (6). Disclosure to sexual partners, family members, close friends and other individuals may result in support and encouragement for oral PrEP use, including pill reminders (7–9). An additional benefit of disclosure to sexual partners is that it could create awareness of oral PrEP and demand for oral PrEP by partners themselves (10). However, in addition to several benefits of disclosure, disclosure may pose challenges as an outcome of oral PrEP being maybe mistaken for HIV treatment, disbelief that HIV prevention pills exist, and oral PrEP use being may be associated with having multiple partners (8–11). Furthermore, non-disclosure of PrEP may make storage of PrEP pills and daily adherence difficult (11).

In this qualitative analysis, we explore oral PrEP disclosure among young women from Durban, South Africa who had initiated oral PrEP while participating in a large contraceptive study (12). In this study, PrEP use was optional as oral PrEP was provided as part of the trial's HIV prevention package. We discuss several aspects related to disclosure including to whom disclosure was made, reactions to disclosure, barriers to disclosure and impact of disclosure on oral PrEP use. Understanding these dynamics of disclosure on oral PrEP use, in this population, could contribute to future PrEP counselling guidelines and strategies, as well as policies for PrEP delivery. This could further facilitate uptake of and adherence to oral PrEP.

## Materials and methods

### Study design

This qualitative research was an exploratory study that was conducted as part of an ancillary study with participants in the Evidence for Contraceptive Options and HIV Outcomes (ECHO) Trial (12).

COREQ (consolidated criteria for reporting qualitative research) guidelines have been used to report the research process below (13).

#### ECHO Trial overview

The ECHO Trial was conducted from 2015 to 2018 at 12 study sites in four African countries and aimed to assess the effect of three contraceptives (intramuscular injectable depot medroxyprogesterone acetate, the copper intrauterine device or the levonorgestrel implant) on HIV incidence. This open-label study enrolled 7,830 women aged 16–35 years with quarterly follow-up for 12–18 months. Detailed study procedures are published elsewhere (12, 14). The study found that there was no substantial difference in HIV risk among those using any of the three contraceptive methods and all three methods were safe and highly effective (12).

#### Oral PrEP provision in the ECHO trial

At the nine South African ECHO Trial sites, oral tenofovir-based PrEP was provided onsite by trial staff as part of the HIV prevention package offered to study participants during the latter eight months (March–October 2018) of the trial. Details regarding the integration and provision of oral PrEP are published (15). At the end of the trial, women who requested to continue using oral PrEP were given a 3-month supply of PrEP and then referred to off-site facilities for ongoing oral PrEP access.

#### Oral PrEP initiation and continuation at the Durban, South Africa, ECHO trial site

At the Durban, South Africa trial site, 861 women were enrolled in the ECHO Trial. Of those women still in follow-up when PrEP became available (*n* = 324), 138 (43%) initiated oral PrEP. Of these, 132 participated in an oral PrEP ancillary study (explored patterns of PrEP use and experience using PrEP), and 87 who continued oral PrEP at study exit were contactable approximately 6-months post study exit regarding ongoing oral PrEP use.

### Post-trial oral PrEP access ancillary study

#### Study setting

At the Durban ECHO Trial site in South Africa, an ancillary qualitative study was conducted in 2021 to explore post-trial PrEP access and use (16). This trial site was in an urban setting, and participants were drawn from the city and surrounding townships.

#### Participants, sampling and recruitment

Women were purposively recruited into the ancillary study and included those who had accessed oral PrEP post-trial exit, those who tried to access oral PrEP and were unable to, and those who had discontinued using oral PrEP. The primary study paper has been published elsewhere (16). We aimed to include 12–15 participants who had initiated oral PrEP during the ECHO Trial and had elected to continue using oral PrEP at study exit and were referred to off-site facilities for ongoing oral PrEP access. Recruitment was conducted telephonically, and only 1 person contacted declined participation as she had relocated. We stopped contacting participants once saturation was reached. For this current manuscript, we explore PrEP disclosure among women enrolled in the post-trial oral PrEP access study.

#### Instrument development

A semi-structured interview guide was developed that explored post-trial PrEP access and use. In addition, it briefly explored disclosure of oral PrEP use, including who the woman disclosed to, barriers to disclosure, reaction of the person(s) to whom the woman had disclosed, and impact of disclosure on PrEP use. Interview questions were developed based on the clinician's experience, the literature, and in consultation with the co-authors who have experience in conducting research including qualitative research and have been involved in various oral PrEP studies.

#### Data collection

Women who met the recruitment criteria for the post-trial oral PrEP access ancillary study were invited to participate in a once off in-depth interview during the period from November to December 2021. Interviews were conducted face to face and in English. Interviews were conducted in a private room at the research site by a female interviewer (IB). Participants were informed that this research was being conducted as part of IB's PhD thesis. IB was a study clinician on the ECHO Trial, responsible for providing clinical care, including oral PrEP to study participants, and was of a similar age to the participants. IB had been working in research for 7 years at the time of the interviews, and had experience in qualitative and quantitative research in clinical trial settings. Rapport was easily established with participants as IB had interacted with participants previously during the study, and provided clinical care at follow-up visits including PrEP counselling and care. Furthermore, in-depth interviews were conducted at the study site where the main ECHO Trial was conducted so participants were familiar with the research site and staff. Techniques including empathy, being kind, and having a non-judgemental attitude assisted with developing rapport. Participants were also advised that they could choose not to answer any questions that might make them uncomfortable or that they did not want to answer. To remain reflexive, IB acknowledged her position and the potential biases that could result, and regularly debriefed with her PhD supervisor post interviews. In addition, CM was not involved in the data collection activities for this study, which allowed her to look at the data without having preconceived ideas. The in-depth interviews were done one-on-one, and lasted approximately 15–30 min and were audio recorded and transcribed verbatim. Transcripts were not shared with women. Saturation had been reached after 13 interviews were conducted. Measures to reduce the risk of COVID-19 transmission were in place at the research site at the time of this research.

#### Data analysis

Data were analysed thematically using NVivo version 10 (QSR International). An initial code list was developed based on a review of some transcripts. Code generation was both inductive and deductive and was informed by the in-depth interview guide as well as participant responses. The initial code list was developed by the interviewer (IB) and subsequently reviewed by a senior (PhD level) qualitative researcher (CM). Codes were discussed, modified and refined to create a final code list for data analysis. This was done as additional transcripts were reviewed and coded. Thematic areas were identified through information repetition, reviewing similarities and differences in discussions, and interpretation of the data, and were informed by the structure of the interview guide and discussions that were elicited by the interviewer (17).

Study findings are reported thematically below. They are supported by quotes and are presented with the participant's age at enrolment into the ECHO Trial and study number.

#### Ethics

This study was approved by the University of the Witwatersrand's Human Research Ethics Committee (Reference: M191155). All participants signed written informed consent to participate in the in-depth interviews. The ECHO Trial was approved by Research Ethics Committees associated with each trial site and the FHI360 ethics review board.

## Results

Of all 13 women included in this study, the age range was 18–25 years, all women were unmarried and most (92%) did not live with their partner. The majority had secondary level education (77%) and did not receive an income of their own (69%) (Table 1).

**Table 1:** Participant characteristics of women at enrolment into the ECHO trial.

Characteristics	Number (%)
Age	
18–19	3 (23)
20–24	8 (62)
25	2 (15)
Marital status	
Unmarried	13 (100)
Education	
Secondary school (complete/incomplete)	10 (77)
Post-secondary school	3 (23)
Lives with partner	
Yes	1 (8)
No	12 (92)
Partner HIV status	
Positive	0 (0)
Negative	9 (69)
Unknown	4 (31)
Earns own income	
Yes	4 (31)
No	9 (69)

We identified four themes and results are presented under each of these themes below.

• Women are willing to disclose PrEP use
• PrEP disclosure provides support for use
• PrEP disclosure increases PrEP uptake
• Challenges associated with PrEP disclosure

### Women are willing to disclose PrEP use

All women disclosed their oral PrEP use to someone. The majority of women reported disclosing oral PrEP use to family members (mostly sisters and/or mothers) (*n* = 9), and their sexual partners (*n* = 8). Some disclosed to their friends (*n* = 5) and/or community members/neighbours (*n* = 2). Most frequent disclosures were made to sisters and sexual partners. Some women reported disclosing PrEP use to multiple individuals, for example, their partner, family members, and friends, while other women reported disclosing to a single individual such as their mother or partner. Disclosures were frequently made to individuals the woman lives with, for example their mother and/or sister.

Interviewer: Did you tell anybody about you taking PrEP?

Participant: Ja [informal for yes], my sister knew … I'm staying with her … she was taking also PrEP.

Interviewer: Okay so both of you were taking PrEP … Okay and what about your partner? Did he know you're taking it?

Participant: Ja [yes] he knew he knew … he was okay [with the participant taking PrEP] he was okay coz [because] he knows PrEP [heard of PrEP] (PID 4, 23 years)

Despite all women disclosing their PrEP use to at least one person, some women reported barriers that hindered them from disclosing oral PrEP use to their sexual partners. These barriers included fears that their partner would assume PrEP tablets are antiretrovirals (ARVs) to treat HIV and that the woman is living with HIV, and fear that their partner will assume the woman is unfaithful and he will no longer trust her.

“I was scared he will say these [PrEP tablets] are tablets for HIV” (PID 3, 22 years)

“I didn't tell him … because I know … he won't trust me, you know how men behave … he will think that I'm doing something behind or there is cheating involved.” (PID 8, 20 years)

One woman also reported having a previous negative experience when disclosing contraceptive use to her partner. She reported that her partner asked her to discontinue the contraceptive and was afraid the same would occur if she disclosed her PrEP use.

Interviewer: Right and did you tell your partner [that you are using PrEP]?

Participant: No I did not.

Interviewer: What made you decide not to tell him?

Participant: I don't know. I did not tell him, uh the other reason why I did not tell him, I tell him about the IUD. He said the IUD is bad … we broke up because he wanted me to stop the IUD (PID 1, 25 years)

### PrEP disclosure provides support for use

Some women reported that disclosing their oral PrEP use led to support and encouragement for PrEP use, and occasionally, reminders for taking PrEP. This support was frequently provided by those living with the woman. One woman reported support for PrEP use by her family:

“People [family members] were like, they were proud of me, they thought that I was like, that was a good thing to do and that that is a mature thing to do.” (PID 2, 22 years)

“She [mother] was happy **…** she was the one happy coz [because] she know [about] PrEP.” (PID 3, 22 years)

For some women, disclosure led to support for pill-taking by family members including providing reminders to take oral PrEP:

“At home they [family members] knew [woman was taking PrEP]. I used to take my pill at 8 o' clock. Even if I'm not at home or maybe I'm sleeping, they'd [family members] wake me up [and they would say, name of participant] “come and take your pills”.” (PID 2, 22 years)

### Prep disclosure increases PrEP uptake

Some women reported that those to whom they had disclosed were also using oral PrEP or showed an interest in using oral PrEP, or in some cases had also elected to use oral PrEP.

Interviewer: Who did you tell [that you were taking oral PrEP]?

Participant: My sister, everybody at home in fact.

Interviewer: What was their response like?

Participant: Some joined me [to use PrEP] … My sisters did join me [to use oral PrEP] and I told one of my friends, she did join me to take PrEP (PID 8, 20 years)

One woman reported that upon disclosure of her oral PrEP use to her sexual partner with whom she shared a child, he began to take her oral PrEP tablets. This woman also felt that “sharing” her oral PrEP tablets with her partner was “caring”.

Interviewer: Who did you tell [that you were taking PrEP]?

Participant: My partner, my baby daddy [partner with whom she shares a child] … Yes I tell [told] him and he was also interested in in them [PrEP tablets]. He also take them [oral PrEP] with me. I say, “No, it's not yours, it's mine” but he take them.

Interviewer: Your boyfriend was sharing your tablets?

Participant: Yes he was sharing it (laughs) … Sharing is caring (laughs) (PID 13, 23 years)

### Challenges associated with PrEP disclosure

Some women reported challenges when disclosing oral PrEP use which included stigma, misconceptions and mistrust, and a lack of awareness of PrEP.

Stigma was associated with disclosure of with PrEP use, for example, one woman reported feeling shame from community members upon disclosing her PrEP use to them.

“Some of them [community members] they was [were] arguing about the PrEP, they don't trust me when I was, when I was telling them I was eating the PrEP. They say there's no PrEP. There's no prevention for HIV … they didn't believe me … I feel so shame [ashamed].” (PID 10, 24 years)

A lack of awareness of oral PrEP led to misconceptions that PrEP pills were ARVs, for example, one woman reported her sisters felt that oral PrEP tablets were ARVs used for HIV treatment and that she should discontinue using her oral PrEP:

“My sisters told me that those pills [PrEP] are for HIV don't drink it.” (PID 5, 18 years)

There was also mistrust and disbelief associated with PrEP. One woman reported that those to whom she disclosed (friends, sisters and sexual partner) felt there was no benefit to taking oral PrEP:

“It's [PrEP] a waste of time. They don't trust it … they didn't believe in PrEP.” (PID 7, 21 years)

While some women reported challenges associated with disclosure, this did not impact PrEP use, for example, one woman reported her partner wanted her to stop taking oral PrEP due to fear of side effects.

Interviewer: Did you experience a negative reaction from anybody else when you were taking PrEP?

Participant: Yes my boyfriend … he was scared about the reaction of the pill [PrEP] what it's going to do in me in my body.

Interviewer: Okay. Did he try to stop you from taking it?

Participant: Yes he did.

Interviewer: And then what did you do?

Participant: I didn't [stop taking PrEP] (PID 6, 25 years)

The woman who felt ashamed due to negative reactions from her community, including disbelief regarding her PrEP use, reported that this did not stop her from continuing with oral PrEP use.

Interviewer: Did that ever make you stop taking your [PrEP] tablets because of what they [community members were] thinking?

Participant: No … I don't like to listen [to] the people when they say the negative things

Interviewer: So, you were still taking yours [PrEP pills]?

Participant: Still. (PID 10, 24 years)

## Discussion

In this analysis that explored disclosure of oral PrEP use among young women in Durban, South Africa that had initiated daily oral PrEP during a contraceptive study, we found that all women had disclosed their oral PrEP use. Many women reported supportive or neutral reactions from those to whom disclosure was made. Barriers that hindered disclosure to sexual partners included fear of the partner assuming oral PrEP is ARVs for HIV treatment and/or that the woman has other sexual partners and is therefore using oral PrEP. Unfavourable reactions to oral PrEP use among women who had disclosed to partners and others included distrust in the efficacy of oral PrEP to prevent HIV, concerns about oral PrEP side effects and the assumption that oral PrEP is treatment for HIV. While negative reactions in general did not impact continued oral PrEP use, positive reactions did occasionally result in support for oral PrEP use, including adherence reminders.

In our study, we found that all women had disclosed oral PrEP use to at least one individual, with some women disclosing to multiple individuals. Similar high rates of disclosure have been observed in other studies (6, 18, 19). In the HPTN 082 study conducted in South Africa and Zimbabwe, 89% of AGYW aged 16–25 years had disclosed oral PrEP use to at least one individual (18). The 3Ps for Prevention Study conducted in South Africa found that 90% of AGYW reported disclosing oral PrEP use to at least one person (6). A study enrolling pregnant and postpartum women from Cape Town, South Africa also found that all women in this study had disclosed oral PrEP use (19). We postulate that the supportive environment of the ECHO Trial may have extended into the uptake of oral PrEP by the study participants and their willingness to disclose. Women were already receiving 3 monthly HIV testing and HIV prevention counselling as part of the trial HIV prevention package and so adding on another method of HIV prevention with counselling may have supported the confidence to disclose in particular if they had already disclosed participation in the trial with regular HIV testing.

Barriers to disclosing oral PrEP use to partners such as anticipating the partner will assume the women has HIV and is taking ARVs for treatment or that the woman is unfaithful and has other sexual partners has been observed in other studies (19–21). In a study that enrolled AGYW from the Eastern Cape in South Africa, women reported that using oral PrEP might result in partner mistrust (i.e., partners would accuse the women of not trusting them) or the partner might accuse the woman of being unfaithful and having sexual relations with other men (20). Negative reactions to oral PrEP use upon disclosure, including the assumption that oral PrEP is HIV treatment, oral PrEP does not exist or does not prevent HIV, and fear of oral PrEP related side effects have been observed in other studies that enrolled AGYW in South Africa (8, 21, 22). This supports the importance of wider community awareness of oral PrEP to avoid or minimize stigma and misinformation (11). The importance of PrEP demand creation including social marketing has been described (5). Mechanisms to drive demand and increase awareness include demand creation campaigns both in schools and communities (23, 24).

Many studies have documented the benefits of disclosure including support for oral PrEP use and providing reminders to take pills (7, 18, 22). In the HPTN 082 study, oral PrEP disclosure was not associated with increased objective adherence, however, women who reported being supported adults and who had disclosed to their parents had higher adherence (18). In the 3Ps study, overall disclosure was not associated with increased PrEP adherence, however, among those 18 years and younger, adolescents who disclosed to a parent had significantly higher adherence (6). An additional consideration to support PrEP use is the use of couples counselling which could address misconceptions and stigma and provide education and support for PrEP use. PrEP counselling should also address the risks of sharing PrEP pills with partners.

Limitations of our study include a small sample size, recall bias as interviews were conducted approximately 3-years post oral PrEP initiation, the lack of adherence data, and participant checking was not done. Furthermore, a theoretical framework was not used for this analysis and the interviews primarily aimed at exploring participant descriptions of post-trial oral PrEP access. We note that our sample included unmarried women only and most women were not aware of their partner's HIV status. There was little probing into reasons for disclosure and on barriers to disclosure, although some data has been reported. Social desirability and power relations could have influenced our findings, resulting in overreporting of disclosure or under reporting of challenges, as women were interviewed by the study clinician.

Despite these limitations, our study findings provide useful information on experiences disclosing oral PrEP use in South Africa at a time when oral PrEP had just recently been introduced for AGYW and general community oral PrEP awareness was rather limited. Furthermore, our study setting and context differed from other PrEP studies that assess disclosure as women enrolled in the ECHO Trial were desiring contraception and oral PrEP was offered as part of the HIV prevention package rather than being an eligibility criterion for study participation. Since this study there have been developments in the field of PrEP with future options for other PrEP formulations [such as the Dapivirine Ring (25) and injectable PrEP options (26, 27)]. However, oral PrEP is still an option and it might take years before long-acting PrEP formulations are widely available, hence understanding patterns of disclosure can provide insights into adherence and support.

## Conclusions and recommendations

An important lesson learnt from our study is that women are willing to disclose their PrEP use to at least one other person if well supported. We found that women mostly had positive experiences disclosing oral PrEP use including support for oral PrEP use from those to whom disclosure was made. Our findings also indicate additional topics that could be included in oral PrEP counselling and support, such as strategies for disclosure of PrEP use and risks of sharing PrEP pills with partners. Furthermore, family members such as mothers and sisters could be leveraged to provide support for PrEP adherence. The importance of community level oral PrEP education and awareness is essential to reduce stigma, myths and misconceptions regarding oral PrEP, and this will be critical for long-acting PrEP products such as injectables and rings.

## Data Availability

The raw data supporting the conclusions of this article will be made available by the authors, without undue reservation.
